# Trends in and Risk Factors for Recurrent *Clostridioides difficile* Infection, New Haven County, Connecticut, USA, 2015–2020

**DOI:** 10.3201/eid2905.221294

**Published:** 2023-05

**Authors:** Chinenye M. Okafor, Paula Clogher, Danyel Olson, Linda Niccolai, James Hadler

**Affiliations:** Yale School of Public Health, New Haven, Connecticut, USA (C.M. Okafor, L. Niccolai, J. Hadler);; Connecticut Emerging Infections Program, New Haven (P. Clogher, D. Olson, L. Niccolai, J. Hadler);; Yale Institute of Global Health, New Haven (L. Niccolai)

**Keywords:** *Clostridioides difficile*, CDI, chronic disease epidemiology, recurrent CDI, recurrent *Clostridioides difficile*, antimicrobial resistance, bacteria, bacterial infections, Connecticut, nosocomial infections, nitrofurantoin, malignancy, United States

## Abstract

Recurrent *Clostridioides difficile* infection (RCDI) causes an increased burden on the healthcare system. We calculated RCDI incidence and identified factors associated with RCDI cases in New Haven County, Connecticut, USA, during 2015–2020 by using data from population-based laboratory surveillance. A subset of *C. difficile* cases had complete chart reviews conducted for RCDI and potentially associated variables. RCDI was defined as a positive *C. difficile* specimen occurring 2–8 weeks after incident *C. difficile* infection. We compared cases with and without RCDI by using multiple regression. RCDI occurred in 12.0% of 4,301 chart-reviewed *C. difficile* cases, showing a U-shaped time trend with a sharp increase in 2020, mostly because of an increase in hospital-onset cases. Malignancy (odds ratio 1.51 [95% CI 1.11–2.07]) and antecedent nitrofurantoin use (odds ratio 2.37 [95% CI 1.23–4.58]) were medical risk factors for RCDI. The 2020 increase may reflect the impact of the COVID-19 pandemic.

*Clostridioides difficile* infection (CDI) is one of the most common causes of healthcare-associated infection (*1*) and can result in serious illness and death (*2*). CDI is classified into 3 types on the basis of epidemiology: healthcare facility–onset (HCFO), community-onset healthcare facility–associated (CO-HCFA), and community-associated (CA) CDI (*1*). Both HCFO and CO-HCFA are healthcare-associated CDIs, differing in where a person is located at symptom onset. Despite evidence of decreasing healthcare-associated CDI cases in the United States, CA-CDI incidence appears to be stable (*3*).

Recurrent CDI (RCDI), defined as an episode of symptom onset and a positive assay result after an episode with a positive assay result in the previous 2–8 weeks (*4*), is associated with increased burden on the healthcare system and increased medical costs (*2*). As many as an estimated 30% of CDI case-patients will experience a first recurrence, with increasing risk for recurrence after the previous episode (*2*). Specific to RCDI, studies have demonstrated that older age and female sex increase the risk for recurrence (*2*). CDI episodes have been shown to have worse outcomes with each subsequent recurring episode (*2*,*5*). Prior studies have described the possible factors that may increase the risk for recurrence as microbiologic factors (*6*–*9*), clinical characteristics (*5*,*10*–*15*), or epidemiologic factors (*15*). Studies mostly limited to older populations also describe social factors, such as living environment, that may be linked to RCDI (*16*) and readmission (*17*), as well as receipt of additional antibiotics (*16*) after CDI. An increasing percentage of cases that are CA-CDI (*3*) could potentially mean an increase in RCDI attributable to the community despite evidence of overall reduction in RCDI cases (*2*).

Determining trends in RCDI and both predisposing and treatment factors associated with recurrence will provide insight into the effectiveness of measures to prevent RCDI, especially given its burden on the healthcare system. Observations from previous studies of increasing cases of RCDI among CA incident cases while RCDI decreases among healthcare-associated incident CDI (*3*) may require a reexamination of the measures for managing CDI among those with CA-CDI. Because of the COVID-19 pandemic and the consequent increase in hospital admissions (*18*), an increase in RCDI is expected among those with healthcare-associated CDI, but the extent and associated factors are largely unexplored. We aimed to describe the sociodemographic characteristics, clinical underlying conditions and medical history, and medication history up to the point of incident CDI of the population with RCDI in New Haven County, Connecticut, USA, during 2015–2020. Additional objectives were to examine trends in RCDI stratified by epidemiologic class (HCFO, CO-HCFA, or CA) and identify possible factors associated with RCDI in the study population. 

## Methods

### Study Population and Design

Our study used data from the Healthcare-Associated Infections Community Interface (HAIC) Program of the Connecticut Emerging Infectious Program collected during January 2015–December 2020. The HAIC program and its CDI surveillance system are described elsewhere (*1*). In brief, the CDI surveillance program, following a common protocol established by Emerging Infectious Program (EIP) sites in other states, monitors the population-based incidence and incidence trends of CDI through active laboratory surveillance of New Haven County residents, regardless of the location of their CDI diagnosis. A RCDI case was defined as a positive laboratory test for CDI in a person with an incident case 2–8 weeks after the defining positive laboratory test in the incident case.

### Epidemiologic Class

The process of assigning epidemiologic class has been described elsewhere (*1*). In brief, a case was classified as either HCFO or community-onset. HCFO was assigned if it was a hospital-onset CDI (positive stool specimen collected >3 days after hospital admission) or long-term care facility (LTCF) onset (positive stool specimen collected in an LTCF or from an LTCF resident admitted to a hospital). Community-onset was defined by a positive stool specimen collected when a person was an outpatient or within 3 days of their hospital admission. Community-onset CDI was further classified into CA if no healthcare facility visit in the prior 12 weeks was reported; all other community-onset cases were considered CO-HCFA (*1*,*3*). CO-HCFA and HCFO cases were considered healthcare-associated CDI.

### Case Selection

A total of 7,023 incident CDI cases occurred during the study period. All CA-CDI and CO-HCFA cases, and a variable (but no lower than 1:10) random selection of HCFO-CDI cases underwent complete chart reviews at EIP sites (*1*). In determining RCDI incidence and associated factors, we excluded 430 cases without a complete chart review (usually because medical charts were unavailable for abstraction after several attempts at retrieval). We then excluded 74 cases for which an epidemiologic class was not determined and 2,218 HCFO cases not selected for chart review. The final denominator for RCDI incidence and associated factors was 4,301 cases.

We compared characteristics of index CDI cases selected for the study of RCDI and those not selected (Appendix Table 1) and observed that a significantly lower percentage of case-patients selected for analysis had HCFO classification (14.2% vs. 86.2%); they also were younger (median age 65 vs. 75 years) and had a lower mortality rate (3.8% vs. 10.5%). Because of the significantly lower percentage of HCFO cases among selected cases, a consequence of including all CA and CO-HCFA CDI cases but only a sample of HCFO cases, we then conducted a comparison of HCFO cases included and excluded in the final analysis; that review showed no significant difference in sex, ethnicity, and mortality rate between selected and excluded HCFO case-patients. However, we noted significantly higher proportions of White persons (77.1% vs. 66.6%), younger persons (median age 73 vs. 75 years), and those who had incident CDI in 2020 (36.0% vs. 5.1%) among included compared with excluded HCFO case-patients (Appendix Table 2).

**Table 1:** Demographic characteristics of persons with incident CDI, stratified by occurrence of recurrent CDI, New Haven County, Connecticut, USA, 2015–2020*

Characteristic	Total	Recurrent CDI, n = 515	No recurrent CDI, n = 3,786	p value
Year of incident CDI				0.002
2015	751 (17.5)	115 (22.3)	636 (16.8)	0.002
2016	744 (17.3)	86 (16.7)	658 (17.4)	NS
2017	685 (15.9)	68 (13.2)	617 (16.3)	NS
2018	789 (18.3)	77 (15.0)	712 (18.8)	0.03
2019	632 (14.7)	68 (13.2)	564 (14.9)	NS
2020	700 (16.3)	101 (19.6)	599 (15.8)	0.03
Age, y, median (IQR)	65.0 (26.0)	70.0 (23.0)	64.0 (26.0)	<0.001
Sex				0.04
M	1,640 (38.1)	175 (34.0)	1,465 (38.7)	
F	2,661 (61.9)	340 (66.0)	2,321 (61.3)	
Race				
White	3,169 (73.7)	419 (81.4)	2,750 (72.6)	<0.001
Black	430 (10.0)	34 (6.6)	396 (10.5)	0.006
Asian, American Indian, or Pacific Islander	38 (0.9)	1 (0.2)	37 (1.0)	NS
Mixed race or unknown	664 (15.4)	61 (11.8)	603 (15.9)	0.02
Ethnicity				
Hispanic	327 (8.4)	31 (6.5)	296 (8.7)	NS
Non-Hispanic	3,556 (91.6)	448 (93.5)	3,108 (91.3)	0.006

*Values are no. (%) except as indicated. CDI, *Clostridioides difficile* infection; IQR, interquartile range; NS, not significant.

**Table 2:** Clinical characteristics of persons with incident CDI, stratified by occurrence of recurrent CDI, New Haven County, Connecticut, USA, 2015–2020*

Characteristic	Total	Recurrent CDI, n = 515	No recurrent CDI, n = 3,786	p value
Epidemiologic class				
HCFO	610 (14.2)	83 (16.1)	527 (13.9)	NS
CO-HCFA	1,234 (28.7)	174 (33.8)	1,060 (28.0)	0.006
CA	2,457 (57.1)	258 (50.1)	2,199 (58.1)	<0.001
Medical history				
Previous CDI	837 (19.5)	101 (19.6)	736 (19.4)	NS
Immunocompromised	1,397 (32.5)	182 (35.3)	1,215 (32.1)	NS
Cerebrovascular accident	320 (7.4)	48 (9.3)	272 (7.2)	NS
Cognitive impairment or dementia	337 (7.8)	49 (9.5)	288 (7.6)	NS
Malignancy	903 (21.0)	144 (28.0)	759 (20.1)	<0.001
HIV without AIDS	32 (0.7)	7 (1.4)	25 (0.7)	NS
Diabetes mellitus	1,024 (23.8)	127 (24.7)	897 (23.7)	NS
Chronic obstructive pulmonary disease	790 (18.4)	88 (17.1)	702 (18.5)	NS
Chronic liver disease	229 (5.3)	27 (5.2)	202 (5.3)	NS
Heart failure	544 (12.7)	67 (13.0)	477 (12.6)	NS
Myocardial infarction	261 (6.1)	39 (7.6)	222 (5.9)	NS
Peripheral vascular disease	213 (5.0)	33 (6.4)	180 (4.8)	NS
Connective tissue disease	184 (4.3)	21 (4.1)	163 (4.3)	NS
Gastrointestinal disease	809 (18.8)	88 (17.1)	721 (19.0)	NS
Peptic ulcer disease	71 (1.7)	8 (1.6)	63 (1.7)	NS
Morbid obesity	149 (3.5)	20 (3.9)	129 (3.4)	NS
Medication history prior to incident CDI				
Proton pump inhibitors	1,487 (39.9)	190 (41.0)	1,297 (39.7)	NS
Histamine 2 receptor blockers	598 (16.1)	85 (18.5)	513 (15.8)	NS
Antibiotics	2,931 (68.2)	375 (72.8)	2,556 (67.5)	0.015
Penicillins	1,033 (24.0)	115 (22.3)	918 (24.3)	NS
Cephalosporins	1,274 (29.6)	179 (34.8)	1,095 (28.9)	0.007
Tetracyclines	191 (4.4)	32 (6.2)	159 (4.2)	0.037
Nitroimidazole	614 (14.3)	88 (17.1)	526 (13.9)	0.052
Nitrofurantoin	99 (2.3)	21 (4.1)	78 (2.1)	0.004
Immunotherapeutic agents	1,707 (39.7)	190 (36.9)	1,517 (40.1)	NS
Steroids	811 (18.9)	101 (19.6)	710 (18.8)	NS
Chemotherapy	334 (7.8)	49 (9.5)	285 (7.5)	NS
Clinical exposures prior to incident CDI				
Admitted	2,194 (51.1)	264 (51.4)	1,930 (51.0)	NS
CDI as reason for admission	1,098 (50.1)	143 (54.4)	955 (49.5)	NS
Emergency department visit	1,579 (37.7)	216 (43.5)	1,363 (36.9)	0.005
Dialysis	191 (4.6)	29 (5.8)	162 (4.4)	NS
Surgery	561 (13.4)	77 (15.5)	484 (13.1)	NS
Death	163 (3.8)	1 (0.2)	162 (4.3)	<0.001
Treatment of incident CDI				
Total no. cases during 2018–2020	2,121	246	1,875	
Received treatment	1,890 (89.1)	218 (88.6)	1,672 (89.2)	NS
Vancomycin	1,344 (63.4)	162 (65.9)	1,182 (63.0)	NS
Metronidazole	479 (22.6)	47 (19.1)	432 (23.0)	NS
Fidaxomicin	28 (1.3)	3 (1.2)	25 (1.3)	NS
Other antibiotic	7 (0.3)	1 (0.4)	6 (0.3)	NS
Stool transplant	9 (0.4)	1 (0.4)	8 (0.4)	NS
Probiotics	383 (18.1)	40 (16.2)	343 (18.3)	NS
>1 treatment course duration	458 (21.6)	55 (22.4)	403 (21.5)	NS

*Values are no. (%) except as indicated. CA, community-associated; CDI, *Clostridioides difficile* infection; CO-HCFA, community-onset healthcare facility–associated; HCFO, healthcare facility–onset; NS, not significant.

### Participant Characteristics 

Medical records abstraction was carried out by trained EIP personnel. For this study, we classified the retrieved information into categories of sociodemographic factors, medication history, medical history, clinical exposures, and treatment received and outcome.

Sociodemographic variables included age, sex, and race (grouped as White; Black; Asian, American Indian, or Pacific Islander; and mixed race or unknown race). Mixed race within available data was a combination of either Black and White race (n = 5) or Black and American Indian race (n = 1). Ethnicity was grouped into non-Hispanic and Hispanic categories. 

Medication history included medications taken within the 12 weeks before a positive incident CDI sample. Medications included proton pump inhibitors, histamine receptor 2 blockers, antibiotics, and immunotherapeutic agents. Immunotherapeutic agents included steroids, chemotherapy agents, and other immunosuppressants. Antibiotics were classified into the different drug classes: penicillin, macrolides, aminoglycosides, cephalosporins, fluoroquinolones, trimethoprim or trimethoprim/sulfamethoxazole, carbapenem, glycopeptide, tetracycline, nitroimidazole, and nitrofurans (Appendix Table 3). 

**Table 3:** Characteristics of participants with recurrent CDI, stratified by epidemiologic class, New Haven County, Connecticut, USA, 2015–2020*

Characteristic	Total	Epidemiologic class			p value
		HCFO, n = 83	CO-HCFA, n = 174	CA, n = 258	
Year of recurrent CDI					<0.001
2015	115 (22.3)	17 (20.5)	36 (20.7)	62 (24.0)	
2016	86 (16.7)	6 (7.2)	31 (17.8)	49 (19.0)	
2017	68 (13.2)	9 (10.8)	22 (12.6)	37 (14.3)	
2018	77 (15.0)	10 (12.1)	31 (17.8)	36 (14.0)	
2019	68 (13.2)	5 (6.0)	29 (16.7)	34 (13.2)	
2020	101 (19.6)	36 (43.4)	25 (14.4)	40 (15.5)	
Age, y, median (IQR)	70.0 (23.0)	74.0 (20.0)	71.0 (23.0)	68.0 (26.0)	0.002
Sex					0.083
M	175 (34.0)	36 (43.4)	61 (35.1)	78 (30.2)	
F	340 (66.0)	47 (56.6)	113 (64.9)	180 (69.8)	
Race					<0.001
White	419 (81.4)	70 (84.3)	134 (77.0)	215 (83.3)	
Black	34 (6.6)	5 (6.0)	13 (7.5)	16 (6.2)	
Asian, American Indian, or Pacific Islander	1 (0.2)	0	1 (0.6)	0	
Mixed race or unknown	61 (11.8)	8 (9.6)	26 (14.9)	27 (10.5)	
Ethnicity					0.007
Hispanic	31 (6.5)	2 (2.5)	14 (8.7)	15 (6.3)	
Non-Hispanic	448 (93.5)	77 (97.5)	147 (91.3)	224 (93.7)	
Medical history					
Previous CDI	101 (19.6)	19 (22.9)	29 (16.7)	53 (20.5)	0.435
Immunocompromised	182 (35.3)	47 (56.6)	71 (40.1)	64 (24.8)	<0.001
Cerebrovascular accident	48 (9.3)	13 (15.7)	19 (10.9)	16 (6.2)	0.024
Cognitive impairment or dementia	49 (9.5)	17 (20.5)	19 (10.9)	13 (5.0)	<0.001
Malignancy	144 (28.0)	22 (26.5)	67 (38.5)	55 (21.3)	<0.001
Medication history					
Proton pump inhibitors	190 (41.0)	47 (58.0)	74 (45.4)	69 (31.5)	<0.001
Histamine 2 receptor blockers	85 (18.5)	19 (23.5)	37 (22.7)	29 (13.4)	0.031
Antibiotics	375 (72.8)	73 (88.0)	145 (83.3)	157 (60.9)	<0.001
Penicillins	115 (22.3)	28 (33.7)	61 (35.1)	26 (10.1)	<0.001
Cephalosporins	179 (34.8)	56 (67.5)	87 (50.0)	36 (14.0)	<0.001
Tetracyclines	32 (6.2)	7 (8.4)	17 (9.8)	8 (3.1)	0.013
Nitroimidazole	88 (17.1)	19 (22.9)	45 (25.9)	24 (9.3)	<0.001
Nitrofurantoin	21 (4.1)	1 (1.2)	10 (5.8)	10 (3.9)	0.012
Immunotherapeutic agents	190 (36.9)	35 (42.2)	68 (39.1)	87 (33.7)	0.292
Clinical exposures					
Admitted	264 (51.4)	57 (68.7)	113 (65.3)	94 (36.4)	<0.001
CDI as reason for admission	143 (54.4)	18 (31.6)	69 (61.6)	56 (59.6)	<0.001
Emergency department visit	216 (43.5)	45 (54.9)	102 (59.0)	69 (28.5)	<0.001
Dialysis	29 (5.8)	9 (11.0)	14 (8.1)	6 (2.5)	0.005
Surgery	77 (15.5)	22 (26.8)	45 (26.0)	10 (4.2)	<0.001

*Values are no. (%) except as indicated. CA, community-associated; CDI, *Clostridioides difficile* infection; CO-HCFA, community-onset healthcare facility–associated; HCFO, healthcare facility–onset; IQR, interquartile range.

Medical history included history of CDI, immunocompromised states (including HIV with or without AIDS; diabetes mellitus; primary immunodeficiency; solid organ transplant, hematopoietic stem cell transplant, or both), cerebrovascular accidents (CVA) (including stroke and transient ischemic attacks), chronic cognitive deficits or dementia, and malignancies (including hematologic and solid organ malignancy with or without metastasis). Treatment variables included vancomycin, metronidazole, fidaxomicin, stool transplant, probiotics, and >1 treatment course duration. 

Clinical data relevant to determining epidemiologic class (clinical exposures) included CDI as reason for admission and whether patients had an emergency department visit, dialysis, or surgery within 12 weeks before sample collection. Because the case report form changed from 2017 to 2018 with respect to treatment variables, only treatment data from 2018–2020 are included in this analysis.

### Primary Outcome

The primary outcome was recurrence of CDI, which we defined as a positive CDI sample using either or both of toxin assay or molecular assay and occurring 2–8 weeks after an episode of incident CDI. We defined incident CDI as a positive diagnostic stool specimen using either or both of toxin assay and molecular assay of any resident of New Haven County who was >1 year of age. We classified CDI cases occurring after 8 weeks of incident CDI as new incident cases and CDI cases occurring within 2 weeks of incident CDI as duplicate cases (*1*). We collected all information on the primary outcome, including information on whether death occurred within 90 days, through medical chart abstraction. We validated mortality data by using the Connecticut death registry database.

### Data Analysis

We calculated annual incidence rates of CDI, excluding RCDI, overall and by epidemiologic class, by using the New Haven County population for each year as a denominator. We calculated incidence rates for RCDI (cases/100 initial cases), overall and by epidemiologic class, by using CDI cases selected for analysis as denominators for each study year. We excluded cases in which the patient died within 2 weeks of incident CDI from the denominator. We compared demographic, clinical, medical, and treatment characteristics of patients with CDI with and without recurrence and then compared distribution of the same characteristics among patients with RCDI, stratified by epidemiologic class. We used the Kruskal-Wallis test for continuous variables and χ^2^ (Fisher exact test if cell frequency was <5) for categoric variables.

Multivariable logistic regression involved the addition of prespecified domains in a sequential manner. We selected covariates for the multivariable regression on the basis of statistical significance at p = 0.05 in the comparison of case-patients with and without RCDI. Multivariable model building used forward elimination, enabling visualization of the effect of each category on the overall model (*19*).

Model 1 encompassed year of incident CDI, age, sex, race, and ethnicity. Race variables were Black compared with White and multiracial or unknown race compared with White. Model 2 includes year of incident CDI, age, sex, race, ethnicity, use of proton pump inhibitors, histamine 2 receptor blockers, antibiotics, penicillin, cephalosporin, tetracycline, nitroimidazole, nitrofuran, and immunotherapy, as well as previous CDI episode, immunocompromised state, CVA, malignancies, chronic cognitive deficit or dementia, admission because of CDI, emergency department visit, dialysis, and surgery. Model 3 encompassed the epidemiologic classes in addition to variables in model 2. Results from the regression models are presented as odds ratio (OR) of RCDI for each covariate and its 95% CI. We conducted all statistical analysis by using SAS version 9.4 (SAS Institute) with 2-tailed tests of significance at α = 0.05 level. Analyses of surveillance data obtained and conducted by Yale EIP staff have been granted a blanket exemption from Yale institutional review board review.

## Results

### Incidence Rates of CDI and RCDI

**Figure 1:**
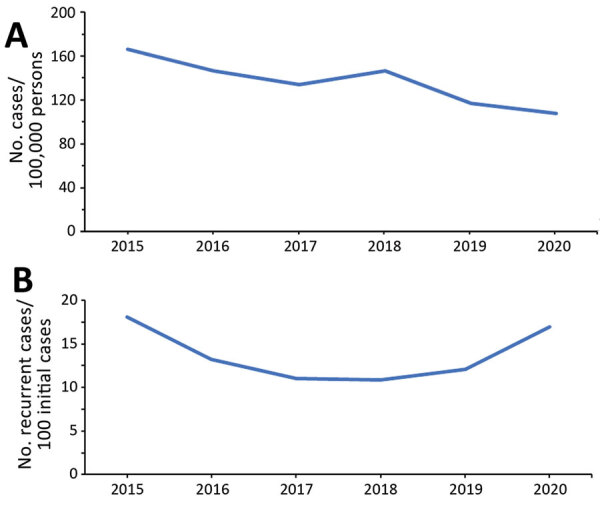
Annual incidence rates of *Clostridioides difficile* infection (CDI) (A) and recurrent CDI (B), New Haven County, Connecticut, USA, 2015–2020. CDI cases were all reported CDI cases in New Haven County. Recurrent CDI cases were available only for cases with complete chart reviews.

A total of 7,023 incident CDI cases occurred during 2015–2020, of which 4,301 cases had a complete chart review. The incidence rate of CDI had a downward trend during the study years, ranging from 165.2 cases/100,000 persons in 2015 to 107.8 cases/100,000 persons in 2020 (median 140.2 cases/100,000 persons). The incidence of RCDI was U-shaped across the study period: 18.1 cases/100 incident cases in 2015, dropping to 13.2–12.1 cases/100 incident cases during 2016–2019, then rising to 16.9 cases/100 incident cases in 2020 (Figure 1).

**Figure 2:**
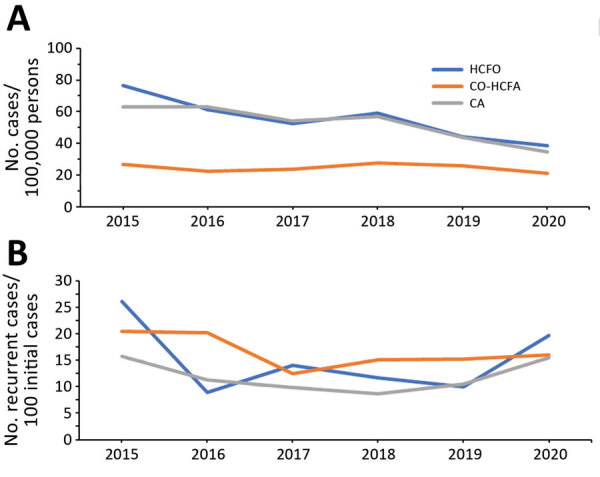
Annual incidence rates of *Clostridioides difficile* infection (CDI) (A) and recurrent CDI (B), by epidemiologic class, New Haven County, Connecticut, USA, 2015–2020. Recurrent CDI cases were available only for cases with complete chart reviews. Epidemiologic class of incident cases was not available for 74 cases because medical records were not available. CA, community-associated; CO-HCFA, community-onset healthcare facility–associated; HCFO, healthcare facility–onset.

When stratified according to epidemiologic class, the incidence of total CDI cases showed a fluctuating pattern but overall decreases in HCFO and CA-CDI over time. The overall U-shaped incidence of RCDI was contributed to mainly by CA and HCFO CDI; the increase in 2020 was largely contributed to by a sharp 37.3% increase in HCFO CDI during 2019–2020 (Figure 2). Throughout the study period, the rate of recurrent CA-CDI cases generally remained consistently below the rate of RCDI among HCFO cases. Overall, 12.0% of 4,301 CDI cases with complete chart reviews had RCDI. By epidemiologic class, the recurrence rate was 13.6% for HCFO-CDI, 14.1% for CO-HCFA-CDI, and 10.5% for CA-CDI.

### Sociodemographic and Clinical Characteristics of Patients with RCDI

Among the 4,301 CDI cases with complete chart reviews, the median age of patients was 65.0 years; 61.9% of patients were female, 73.7% were White, and 8.4% were Hispanic. RCDO occurred in 12.0% of cases. A significantly higher proportion of patients with RCDI were older (median age 70.0 vs. 64.0 years), female (66.0% vs. 61.3%), White, and non-Hispanic and had incident CDI cases classified as healthcare-associated CDI (HCFO and CO-HCFA) (Table 1, 2). The only underlying condition that was significantly higher among case-patients with RCDI was a history of malignancy. Among antecedent medicines, only antecedent antibiotic use in aggregate, and specifically, cephalosporins, tetracyclines, and nitrofurans, were significantly more frequent among patients with RCDI. No specific treatment for the incident CDI case was significant, so specific treatment was not included in modeling (Table 2).

When we compared cases with RCDI by epidemiologic class of the incident CDI episode, we noted that a significantly higher proportion in HCFO case-patients were older (median age 74.0 years), White, and non-Hispanic (Table 3). This group also had a significantly higher proportion of persons with CVA and immunocompromised states, including diabetes mellitus. Proportions of persons with malignancies, a history of nitrofurantoin use, incident CDI as reason for admission, and emergency department use were significantly higher among the CO-HCFA CDI class.

### Factors Associated with RCDI

The multivariable models indicated that Black race when compared to White race and year of incident CDI (except 2020) when compared to 2015 were associated with lower odds of RCDI (Table 4). The associations for incident CDI occurring in 2016 (compared with 2015) was attenuated in the final model, but results remained significant for 2017 (OR 0.43 [95% CI 0.26–0.73]), 2018 (OR 0.60 [95% CI 0.37–0.97]), and 2019 (OR 0.50 [95% CI 0.30–0.84]) (all compared with 2015) and for Black race compared with White race (OR 0.50 [95% CI 0.30–0.83]). When year of incident CDI was regressed as an ordinal variable (i.e., 2015 to 2020 represented by numbers 1 to 6 and regressed as is), we observed no statistical significance in the univariate analysis and multivariable results for all 3 models. In the final model, case-patients with malignancies were 51% more likely to have RCDI (OR 1.51 [95% CI 1.11–2.07]), whereas nitrofurantoin use before an incident CDI episode had 137% higher odds for RCDI (OR 2.37 [95% CI 1.23–4.58]).

**Table 4:** Factors associated with recurrent CDI identified in 3 models, New Haven County, Connecticut, USA, 2015–2020*

Factor	OR (95% CI)		
	Model 1*	Model 2†	Model 3‡
Year of recurrent CDI			
2016	0.73 (0.54–1.00)†	0.68 (0.44–1.05)	0.68 (0.44–1.06)
2017	0.59 (0.42–0.83)†	0.43 (0.26–0.72)‡	0.43 (0.26–0.73)§
2018	0.69 (0.50–0.96)†	0.59 (0.37–0.95)‡	0.60 (0.37–0.97)§
2019	0.69 (0.49–0.97)†	0.50 (0.30–0.84)‡	0.50 (0.30–0.84)§
2020	0.98 (0.72–1.32)	0.72 (0.46–1.12)	0.73 (0.46–1.16)
Age	1.02 (1.01–1.02)†	1.01 (1.00–1.02)	1.01 (1.00–1.02)
Female sex	1.20 (0.98–1.46)	1.22 (0.92–1.63)	1.22 (0.92–1.63)
Race or ethnicity			
Black	0.61(0.42–0.89)†	0.50 (0.31–0.83)‡	0.50 (0.30–0.83)§
Asian/American Indian/Pacific Islander	0.27 (0.04–1.98)	<0.01 (<0.01 to >999.99)	<0.01 (<0.01 to >999.99)
Mixed/Unknown race	0.97 (0.54–1.75)	1.36 (0.58–3.20)	1.36 (0.58–3.19)
Hispanic	0.90 (0.51–1.58)	0.64 (0.28–1.50)	0.65 (0.28–1.51)
Medication history			
Proton pump inhibitors		0.85 (0.64–1.13)	0.85 (0.64–1.13)
Histamine 2 receptor blockers		0.98 (0.68–1.41)	0.97 (0.67–1.39)
Antibiotics		1.21 (0.80–1.81)	1.21 (0.81–1.81)
Penicillins		1.03 (0.74–1.44)	1.02 (0.73–1.42)
Cephalosporins		1.13 (0.81–1.57)	1.10 (0.79–1.54)
Tetracyclines		1.05 (0.60–1.85)	1.05 (0.60–1.85)
Nitroimidazole		0.93 (0.64–1.35)	0.93 (0.65–1.34)
Nitrofurantoin		2.38 (1.23–4.59)‡	2.37 (1.23–4.58)§
Immunotherapeutic agents		0.86 (0.62–1.18)	0.85 (0.62–1.17)
Clinical history			
Previous CDI		1.03 (0.74–1.44)	1.03 (0.74–1.44)
Immunocompromised		1.20 (0.89–1.61)	1.19 (0.88–1.60)
Cerebrovascular accident		0.91 (0.58–1.43)	0.90 (0.57–1.41)
Cognitive Impairment or dementia		0.91 (0.58–1.42)	0.91 (0.58–1.42)
Malignancy		1.52 (1.11–2.08)‡	1.51 (1.11–2.07)§
Clinical exposures			
CDI as reason for admission		1.48 (1.10–2.01)‡	1.46 (1.07–12.01)§
Emergency department visit		1.32 (1.00–1.76)	1.28 (0.95–1.71)
Dialysis		1.45 (0.85–2.47)	1.44 (0.85–2.46)
Surgery		1.14 (0.80–1.64)	1.11 (0.77–1.59)
Epidemiologic class			
CO-HCFA			1.10 (0.74–1.63)
CA			0.93 (0.61–1.42)

*Model 1 contains year of incident CDI diagnosis, age, sex, race, and ethnicity. Model 2 contains, in addition to model 1 variables, use of proton pump inhibitors, histamine 2 receptor blocker, antibiotics, penicillin, cephalosporin, tetracycline, nitroimidazole, nitroimidazoles, nitrofurans, immunotherapeutic agents, previous CDI, immunocompromised states, cerebrovascular accident, cognitive impairment or dementia, malignancy, admission because of CDI, emergency department visit, dialysis, and surgery. Model 3 contains, in addition to variables in model 2, the epidemiologic class of cases. CA, community-associated; CDI, *Clostridioides difficile* infection; CO-HCFA, community-onset healthcare facility–associated; OR, odds ratio. †Statistically significant in the first model. ‡Statistically significant in the second model. §Statistically significant in the final model.

## Discussion

We observed a general reduction in CDI incidence over the entire study period and an increase in incidence rates of RCDI from 2019 to 2020 despite initial decreases from 2015 to 2018. When stratified by epidemiologic class of CDI, the increase in RCDI was largely attributable to an increase in recurrent HCFO-CDI in 2020 and, to a lesser extent, small, gradual increases in recurrence rates for CA-CDI during 2018–2020. After adjusting for year of incident CDI, sex, age, race, ethnicity, medication history, underlying conditions, clinical exposures, treatment, and epidemiologic class of incident CDI, we observed significantly increased risk for RCDI among case-patients who were admitted for non-CDI reasons and those with a history of malignancy and nitrofurantoin use.

This study highlights that the observed decrease in incidence of RCDI from 2015 to 2018 adds to the published literature by emphasizing a lower rate of RCDI overall (*12*) and in all epidemiologic classes of RCDI (*3*) and reflecting a continued decrease in RCDI from before 2015 (*20*). The decrease in RCDI may be attributable to current measures for managing and preventing RCDI, including restrictions on prescription of fluroquinolones (*3*,*21*), infection control measures (*22*), and possibly adherence to approved treatment guidelines (*23*), being effective enough to produce a notable reduction in RCDI rates. Studies of RCDI rates in 2020 are scarce; thus, this study provides early possible evidence of increasing rates of RCDI from 2019 to 2020. Although the CDI case report form changed in 2019 (*24*) and improvements were made in the laboratory techniques for detection of CDI, those factors would not explain the increase in RCDI occurring from 2019 to 2020 without a similar pattern occurring from 2018 to 2019. Given that the major distinguishing factor between the year 2019 and 2020 was the COVID-19 pandemic, the increase in 2020 could be related to that. Of note, a recent US study found that, although the incidence of several healthcare-associated infections increased during periods of high COVID hospital admissions, incident CDI did not (*25*). However, that study did not specifically look at RCDI.

Prior studies have suggested that persons with a history of malignancy (*26*), prior antibiotic use (*21*,*27*), increasing age (*23*,*28*), female sex (*28*), and White race (*28*) have an increased risk for CDI recurrence. Although our study shows that these factors are important predictors of RCDI, adjusting for patient-level risk factors nullified the significance of age and sex in predicting RCDI. Significant predictors of RCDI in the final model were year of incident CDI, White race, a history of malignancy, admission for incident CDI care, and nitrofurantoin use before incident CDI. Nitrofurantoin is prescribed for conditions such as urinary tract infection, more commonly used among older populations (*29*,*30*), and may be in part a marker for older persons with a longer pre-CDI history of antibiotic use than we measured. In addition, the finding of malignancy as the main underlying diagnosis associated with RCDI could reflect an ongoing need for antibiotic therapy, cytotoxic chemotherapy, or both, which could increase the risk for RCDI (*14*). We can draw parallels between the significant risk factors for RCDI from this study and some known factors that lead to severe COVID-19 disease: older age (*31*,*32*) and a history of malignancy (*33*). Taken together, those factors could, at least in part, explain the increase in RCDI in 2020, in addition to antibiotic prescription in treatment of COVID-19 (*34*,*35*). Further understanding of the effect of COVID-19 on the incidence of CDI, which at this time is only an ecologic association, and other factors that may have influenced increased rates of RCDI during the pandemic are needed to determine whether COVID-19–specific interventions are needed to prevent RCDI in the pandemic era.

Although nitrofurantoin is known to have minimal effect on bowel flora, given that it concentrates in the urinary tract and has low serum concentration (*36*), this study shows a significant relationship between nitrofurantoin use and increased risk for RCDI. This observation could be attributable to the higher rates of prescription of multiple antibiotics to older populations (*37*,*38*). Other studies have found a tendency for inappropriate antibiotic use in LTCFs (*39*–*41*). However, after controlling for overall antibiotic use, nitrofurantoin use remained a significant risk factor for RCDI. Further studies into possible explanatory factors are needed, because currently available clinical factors do not completely explain increased risk for RCDI (*42*).

The first limitation of this study is that the surveillance system does not collect information on antibiotic or other medication use once a patient with incident CDI is admitted, other than whatever treatment regimen was initiated for treatment of the incident CDI infection. The collected information on medication use was limited to the 12 weeks before incident CDI diagnosis, which does not account for possible effects of antibiotics taken before that period. Nitrofurantoin use, for example, could conceivably be a marker for patients with recurrent urinary tract infections who received multiple rounds of antibiotics in the past. Second, we were only able to look at the treatment regimen in a standardized way for the years 2018–2020, limiting our power to find significance, and then only for the initial treatment ordered, not changes to it. Third, because symptom information is not routinely collected for patients with recurrent cases, some of those cases could be misclassified if the reason for the patient’s RCDI qualifying laboratory test were to assess test of cure or colonization status. In addition, the protocol for chart reviews used by HAIC allows for a limited random sampling of 1 in 10 HCFO cases, which could provide a potential point of bias given that information on CDI recurrence is unavailable for unsampled HCFO case-patients. Further, the review and modification of the CDI case report form over the years has helped improve reporting of patient-level characteristics of CDI cases. However, those changes have also produced variations in the case report form and the way information is collected, something particularly noticeable in the case report form change in 2019, which saw the inclusion of information such as route of medication for treatment of previous CDI and number of courses of treatment received, factors that could possibly be important predictors of recurrence. Last, we were unable to adjust for type of test; a previous study found that incident CDI case-patients identified by a common testing algorithm in which confirmation was done by nucleic acid amplification test (NAAT) were less likely to have RCDI than case-patients identified through toxin testing (*43*), presumably because NAAT may be more likely to detect colonization than toxin testing. If testing of index CDI case-patients that used algorithms that were less likely to be falsely positive (identifying colonization) over time, then the increase in RCDI risk we observed from 2019 to 2020 might be partly attributable to that tendency. However, stratifying the risk for RCDI by tests that included NAAT versus those that did not made no difference in the magnitude or statistical significance of the increase.

Over the study period, a notable decline occurred in the incidence of RCDI, until 2020, when a sharp increase occurred. The initial reduction in RCDI incidence over time may reflect the effectiveness of measures for management of CDI, prevention of RCDI, or both. The increased risk for CDI recurrence in patients with a history of using nitrofurantoin, a drug commonly prescribed for urinary tract infections in older persons, and patients with malignancies reflects a particularly vulnerable population that requires targeted programs to prevent RCDI.

Appendix Additional information about trends in and risk factors for recurrent *Clostridioides difficile* infection, New Haven County, Connecticut, USA, 2015–2020.
