# Supplementary material for: Trends in and Risk Factors for Recurrent Clostridioides difficile Infection, New Haven County, Connecticut, USA, 2015–2020
Source: Emerg Infect Dis. 2023 May;29(5):877–87. doi: 10.3201/eid2905.221294 (PMC10124664; doi:10.3201/eid2905.221294)
Supplement: Appendix — Additional information about trends in and risk factors for recurrent Clostridioides difficile infection, New Haven County, Connecticut, USA, 2015–2020. [file 22-1294-Techapp-s1.pdf]

Article DOI: https://doi.org/10.3201/eid2905.221294

Trends in and Risk Factors for Recurrent *Clostridioides difficile* Infection, New Haven County, Connecticut, USA, 2015–2020

Appendix

Appendix Table 1. Comparison of Sociodemographic Characteristics of Incident Cases Selected for Analysis with Excluded Cases.

Variable (%)	Total (N, %)	Selected for Analysis (N=4301)	Not Selected for Analysis (N=2722)	p-value
Year				<0.001
2015	1429 (20.4)	751 (17.5)	678 (24.9)	
2016	1259 (17.9)	744 (17.3)	515 (18.9)	
2017	1148 (16.4)	685 (15.9)	463 (17.0)	
2018	1256 (17.9)	789 (18.3)	467 (17.2)	
2019	999 (14.2)	632 (14.7)	367 (13.5)	
2020	932 (13.3)	700 (16.3)	232 (8.5)	
Age (Median. IQR)	68.0 (25.0)	65.0 (26.0)	73.0 (23.0)	<0.001
Sex				<0.001
Male	2858 (40.7)	1640 (38.1)	1218 (44.8)	
Female	4165 (59.3)	2661 (61.9)	1504 (55.3)	
Race				<0.001
White	4958 (70.6)	3169 (73.7)	1789 (65.7)	
Black	751 (10.7)	430 (10.0)	321 (11.8)	
Asian/American Indian/Pacific Islanders	50 (0.7)	38 (0.9)	12 (0.4)	
Mixed/Unknown race	1264 (18.0)	664 (15.4)	600 (22.0)	
Ethnicity				0.010
Hispanic	476 (7.8)	327 (8.4)	149 (6.6)	
Non-Hispanic	5662 (92.3)	3556 (91.6)	2106 (93.4)	
Mortality	401 (6.1)	163 (3.8)	238 (10.5)	<0.001
Epidemiological Class				<0.001
HCFO	2852 (41.3)	610 (14.2)	2242 (86.2)	
CO-HCFA	1264 (18.3)	1234 (28.7)	30 (1.2)	
CA	2708 (39.2)	2457 (57.1)	251 (9.7)	

HCFO=healthcare facility onset, CO-HCFA=community-onset healthcare facility associated, CA=community-associated

Appendix Table 2. Comparison of Sociodemographic Characteristics of Incident HCFO Cases Selected for Analysis with Excluded Incident HCFO cases

Variable (%)	Total (N, %)	HCFO Cases Selected for Analysis (N=610)	HCFO Cases Not Selected for Analysis (N=2242)	p-value
Year				<0.001
2015	657 (23.0)	82 (13.4)	575 (25.7)	
2016	527 (18.5)	79 (13.0)	448 (20.0)	
2017	451 (15.8)	74 (12.1)	377 (16.8)	
2018	507 (17.8)	100 (16.4)	407 (18.2)	
2019	378 (13.3)	55 (9.0)	323 (14.4)	
2020	332 (11.6)	220 (36.1)	112 (5.0)	
Age (Median. IQR)	74.0 (22.0)	73.0 (22.0)	75.0 (22.0)	0.009
Sex				0.215
Male	1288 (45.2)	289 (47.4)	999 (44.6)	
Female	1564 (54.8)	321 (52.6)	1243 (55.4)	
Race				<0.001
White	1962 (68.8)	470 (77.1)	1492 (66.6)	
Black	367 (12.9)	78 (12.8)	289 (12.9)	

Variable (%)	Total (N, %)	HCFO Cases Selected for Analysis (N=610)	HCFO Cases Not Selected for Analysis (N=2242)	p-value
Asian/American Indian/Pacific Islanders	13 (0.5)	3 (0.5)	10 (0.5)	
Mixed/Unknown race	510 (17.9)	59 (9.7)	451 (20.1)	
Ethnicity				0.283
Hispanic	159 (6.4)	43 (7.4)	116 (6.1)	
Non-Hispanic	2317 (93.6)	540 (92.6)	1777 (93.9)	
Mortality	309 (12.0)	72 (11.8)	237 (12.0)	0.877

HCFO = Healthcare facility onset

Appendix Table 3. Classification of Antibiotics by Class

Class Name	Antibiotic Variables
Penicillins	Amoxicillin, Amoxicillin/Clavulanic Acid, Ampicillin, Ampicillin/Sulbactam, Penicillin, Piperacillin-Tazobactam
Aminoglycosides	Amikacin, Gentamicin, Tobramycin
Macrolides	Azithromycin, Clarithromycin, Clindamycin, Daptomycin
Cephalosporins	Cefazolin, Cefixime, Cefepime, Cefotaxime, Cefoxitin, Cefpodoxime, Ceftaroline, Ceftazidime, Ceftazidime/avibactam, Ceftizoxime, Ceftolozane/tazobactam, Ceftriaxone, Cefuroxime, Cephalexin
Fluoroquinolones	Ciprofloxacin, Delafloxacin, Levofloxacin, Moxifloxacin
TMP/TMP-SMZ	Trimethoprim, Trimethoprim-Sulfamethoxazole
Glycopeptides	Dalbavancin, Vancomycin, Oritavancin, Telavancin
Carbapenems	Doripenem, Ertapenem, Imipenem/cilastatin, Meropenem, Meropenem/vaborbactam
Tetracyclines	Doxycycline
Nitroimidazoles	Metronidazole
Nitrofurans	Nitrofurantoin
Rifamycin	Rifaximin
